# The Transcription Factor ArcA Modulates *Salmonella*’s Metabolism in Response to Neutrophil Hypochlorous Acid-Mediated Stress

**DOI:** 10.3389/fmicb.2019.02754

**Published:** 2019-12-05

**Authors:** Coral Pardo-Esté, Juan Castro-Severyn, Gabriel I. Krüger, Carolina Elizabeth Cabezas, Alan Cristóbal Briones, Camila Aguirre, Naiyulin Morales, Maria Soledad Baquedano, Yoelvis Noe Sulbaran, Alejandro A. Hidalgo, Claudio Meneses, Ignacio Poblete-Castro, Eduardo Castro-Nallar, Miguel A. Valvano, Claudia P. Saavedra

**Affiliations:** ^1^Laboratorio de Microbiología Molecular, Departamento de Ciencias Biológicas, Facultad de Ciencias de la Vida, Universidad Andres Bello, Santiago, Chile; ^2^Centro de Bioinformática y Biología Integrativa, Facultad de Ciencias de la Vida, Universidad Andres Bello, Santiago, Chile; ^3^Laboratorio de Patogenesis Bacteriana, Facultad de Medicina, Universidad Andres Bello, Santiago, Chile; ^4^Centro de Biotecnología Vegetal, Facultad de Ciencias de la Vida, Universidad Andres Bello, Santiago, Chile; ^5^FONDAP Center for Genome Regulation, Universidad Andres Bello, Santiago, Chile; ^6^Wellcome-Wolfson Institute for Experimental Medicine, Queen’s University Belfast, Belfast, United Kingdom; ^7^Millennium Institute on Immunology and Immunotherapy, Departamento de Ciencias Biológicas, Facultad de Ciencias de la Vida, Universidad Andres Bello, Santiago, Chile

**Keywords:** ArcA, neutrophils, *Salmonella*, transcription factor, hypochlorous acid

## Abstract

*Salmonella* Typhimurium, a bacterial pathogen with high metabolic plasticity, can adapt to different environmental conditions; these traits enhance its virulence by enabling bacterial survival. Neutrophils play important roles in the innate immune response, including the production of microbicidal reactive oxygen species (ROS). In addition, the myeloperoxidase in neutrophils catalyzes the formation of hypochlorous acid (HOCl), a highly toxic molecule that reacts with essential biomolecules, causing oxidative damage including lipid peroxidation and protein carbonylation. The bacterial response regulator ArcA regulates adaptive responses to oxygen levels and influences the survival of *Salmonella* inside phagocytic cells. Here, we demonstrate by whole transcriptomic analyses that ArcA regulates genes related to various metabolic pathways, enabling bacterial survival during HOCl-stress *in vitro*. Also, inside neutrophils, ArcA controls the transcription of several metabolic pathways by downregulating the expression of genes related to fatty acid degradation, lysine degradation, and arginine, proline, pyruvate, and propanoate metabolism. ArcA also upregulates genes encoding components of the oxidative pathway. These results underscore the importance of ArcA in ATP generation inside the neutrophil phagosome and its participation in bacterial metabolic adaptations during HOCl stress.

## Introduction

The pathogen *Salmonella enterica* serovar Typhimurium (*S.* Typhimurium) can adapt to different environments by rapidly modulating gene expression (Hébrard et al., 2011). Critically to its infection cycle, this bacterium can survive the microbicidal action of immune cells, including neutrophils (Ibarra and Steele-Mortimer, 2009; Ruby et al., 2012; Behnsen et al., 2015).

*Salmonella* promotes its own internalization to the host via several effector proteins associated with *Salmonella* Pathogenicity Island 1 (SPI-1), namely: SipA, SipC, and SptP (LaRock et al., 2015). Once engulfed, the bacterium encounters the oxidative burst, a process in which oxygen is reduced to form superoxide radicals and other toxic reactive oxygen species (ROS). Neutrophils possess two enzymes at play: NADPH oxidase, which initiates the production of O_2_^–^, and myeloperoxidase (MPO), which produces hypochlorous acid (HOCl) (Winterbourn and Kettle, 2013).

Hypochlorous acid is a highly toxic compound that rapidly reacts with functional groups in cysteine, methionine, lysine, histidine, and tyrosine residues, forming mono- and dichloro-amines (Hawkins and Davies, 2002; Beavers and Skaar, 2016). It also targets DNA and lipids, leading to genome-wide mutations, protein inactivation, and membrane perturbations (Pattison and Davies, 2001). Major changes in the bacterial metabolite profile occur as early as after 5 min of HOCl-induced stress, including major shifts in fatty acids, amino acids, and other organic acids such as acetic and formic acid (Drazic et al., 2015).

Metabolic adjustments are essential for bacterial survival. *S.* Typhimurium can adapt *in vivo* according to the infected cell type or *in vitro* to the components of the culture medium (Eriksson et al., 2003; Wang et al., 2010; Hébrard et al., 2011; Srikumar et al., 2015). For instance, the bacterium does not require a complete TCA cycle for efficient replication in epithelial cells and macrophages (Garcia-Gutierrez et al., 2016), and the bacterial cell activates the use of lipid metabolism as an energy source (Diacovich et al., 2017). Biosynthesis of aromatic amino acids, purine, LPS core, O-antigen, and enzymes involved in glycolysis and the TCA cycle, mannose utilization and oxidative phosphorylation are relevant pathways for efficient infection (Chaudhuri et al., 2009), and the most up-regulated functional categories of *S.* Typhimurium genes within macrophages are involved in carbohydrate and amino acid metabolism (Srikumar et al., 2015).

Aerobic respiration control (ArcA) is one of the main transcriptional regulators of the metabolic shift from anaerobic to aerobic conditions and the control of enzymatic defenses against ROS (Evans et al., 2011). ArcA regulates key genes for *Salmonella* survival in response to anaerobiosis, aerobiosis, low oxygen, hydrogen peroxide, and life within phagocytic cells (Shalel-Levanon et al., 2005; Evans et al., 2011; Morales et al., 2013; Park et al., 2013; Jiang et al., 2017; Pardo-Esté et al., 2018; Wang et al., 2018). ArcA also regulates membrane permeability in response to ROS generated by H_2_O_2_ and HOCl, specifically by repressing the expression of multiple porin genes such as *bhsA, ompD, ompC, ompS2, ompF*, and *ompW* (Calderon et al., 2011; Gebendorfer et al., 2012; Morales et al., 2012; Ipinza et al., 2014). Inside macrophages and neutrophils, ArcA modulates the expression of *ompW, ompD*, and *ompF* (Pardo-Esté et al., 2018).

In this study, we determined the role of ArcA in *Salmonella* Typhimurium under HOCl stress. Whole transcriptome analyses revealed a direct relation between ArcA and metabolism modulation under 1 mM NaOCl treatment. Additionally, as neutrophils are phagocytes that use HOCl as their main ROS-related microbicide, we determined gene expression levels in key participants of biosynthetic pathways and found that ArcA is also involved in the bacterial transcriptional response to the environment encountered inside these cells. We demonstrated that transcription level values present mostly a positive correlation in each condition evaluated, underlining the role played by ArcA during the transcriptional response to HOCl in general. ArcA also reduces the magnitude of bacterial oxidative damage inside neutrophils, suggesting an important role for ArcA in the ability of *S.* Typhimurium to survive HOCl stress during its infective cycle.

## Materials and Methods

### Ethics Statement

Animals were treated following the recommendations in the Guide for the Care and Use of Laboratory Animals of the US National Institutes of Health, and the protocol was approved by the Bioethics Committee of Universidad Andrés Bello, Protocol 06/2016 within the framework of FONDECYT Grant #1160315.

### Bacterial Strains and Growth Conditions

The Typhimurium 14028s parental strain and Δ*arcA* were maintained on LB agar plates in aerobiosis unless otherwise indicated. Cells were grown aerobically with shaking in LB medium at 37°C until reaching an OD_600_ of 0.4. *S. enterica* serovar Typhimurium 14028s Δ*arcA*:*aph* strain was obtained previously (Calderon et al., 2011).

### Mouse Bone-Marrow-Derived Neutrophils

C57BL/6 female mice (7–8 weeks old) were used to obtain bone-marrow-derived neutrophils (BMDNs). Mice were kept in plastic cages in a temperature-controlled environment (22–24°C). To prepare mouse neutrophils, bone marrow was extracted as previously described (Swamydas and Lionakis, 2013) and neutrophils were obtained using the mouse Neutrophil Isolation Kit (Milenybiotec) according to the manufacturer’s instructions. This resulted in the isolation of an average of 800,000 neutrophils/ml with around 85% viability; these were positive for CD11b and Ly6G, as determined by flow cytometry. The viability of neutrophils was also monitored throughout the experiments by trypan blue staining.

### Total RNA Extraction From NaOCl-Treated Bacteria and Infected BMDNs

For the total RNA extraction from *in vitro* NaOCl-treated bacteria, overnight cultures of *S.* Typhimurium 14028s and Δ*arcA* were diluted (1:100) and grown to OD_600_ ∼0.4, at which point they were exposed to 1 mM of NaOCl for 20 min before the extraction. Total RNA extraction from infected neutrophil-recovered bacteria was carried out as previously described (Pardo-Esté et al., 2018). Briefly, 10^7^ bacteria/ml grown in microaerophilic conditions were incubated with BMDNs for 3 h. At 1 and 3 h post-infection (pi), cells were harvested, washed twice with PBS, and lysed with sodium deoxycholate (0.5% w/v in PBS), one sample was used as a bacterial viability control and was plated on LB plates. Bacteria were recovered after 1 mM of NaOCl treatment and BMDN infection. RNA extraction from bacteria in both experiments was carried out using the acid–phenol method (Koronakis and Hughes, 1988). RNA was suspended in 30 μl of nuclease-free water and stored at −80°C until used. The integrity of the RNA was assessed by 1.0% agarose gel electrophoresis, and its concentration and quality were verified spectrophotometrically by the OD_260__/__280_ ratio. The RNA was treated with 2 U of DNase I (Roche) for 1 h to remove contaminant DNA. To ensure no carry-over DNA in the samples, we routinely performed PCR amplifications using primers for bacterial 16s RNA and found no product using the RNA extract as a template.

### RNA-Seq Analysis

Total RNA extracted from *in vitro* NaOCl-treated bacteria was depleted of rRNAs, used for cDNA library preparation, and sequenced by Macrogen Inc. (Seoul, South Korea). The TruSeq mRNA Library Prep Kit (Illumina, Inc.) was used, and cDNA was sequenced on an Illumina HiSeq 2500 platform. Two independent libraries (150 bp, single-end) were constructed for each sample (strain – treatment), corresponding to two biological replicates. An average of 9.5 million reads per sample were obtained, representing a depth of 315X. Quality control of raw data was carried out using FastQC v0.11.8 (Andrews, 2010) and filtering and trimming with PRINSEQ v0.20.4 (Schmieder and Edwards, 2011) with 100 bp, 0 N, and < Q20 thresholds. Gene expression levels were estimated by mapping reads against the *S. enterica* subsp. *enterica* serovar Typhimurium strain 14028s reference genome (GenBank: GCA_000022165.1) with Bowtie2 v2.3.5 (Langmead and Salzberg, 2012). The counts of reads that map against *Salmonella* ORFs were obtained using HTSeq v0.11.2 (Anders et al., 2015). The resulting matrix of counts was used to estimate differential gene expression using a normalization method implemented in the edgeR Bioconductor R Package (Robinson et al., 2010). First, we determined the response of each strain to NaOCl treatment by performing, separately, an analysis of differential gene expression in response to stress. From these data, we performed a second step, filtering both data sets-obtained previously with the genes that changed their expression in the mutant vs. the parental strain in control conditions. Third, we matched these data sets of genes to identify those that are only defendant of ArcA regulation under NaOCl stress. Expression values represent log2 after FC ± 2 and FDR ≤ 0.1 filters were applied. RNA-seq data is available in the NCBI SRA database under accession numbers SRR9188680 and SRR9188681 (Bioproject PRJNA357075). GO/KEGG enrichment analysis was carried out using the ClueGO plug-in (Bindea et al., 2009) via Cytoscape (Shannon et al., 2003).

### qRT-PCR

RNA extracted from bacteria recovered from infected neutrophils was reverse transcribed into cDNA at 37°C for 1 h in 25 μl of a mixture containing 2.5 pmol of Random Primers (Invitrogen), 10 μl template RNA (5 mg), 0.2 mM dNTPs, 1 μl sterile water, 4 μl of 5 × buffer (250 mM Tris–HCl pH 8.3, 375 mM KCl, 15 mM MgCl_2_, and 10 mM DTT), and 200 U of reverse transcriptase (Invitrogen). The primers used for qRT-PCR are listed in Supplementary Table S1. The relative quantification of each transcript was performed using the Brilliant II SYBR Green QPCR Master Reagent and the Mx3000P detection system (Stratagene). The qRT-PCR mixture (20 μl) contained 1 μl of the cDNA template and 120 nM of each primer. The qRT- PCR was performed under the following conditions: 10 min at 95°C followed by 40 cycles of 30 s at 95°C, 45 s at 58°C, and 30 s at 72°C. Fold-change expression of target genes, normalized by the expression of a suitable gene selected in these experimental conditions were calculated as described (Pfaffl, 2011). *talB* gene expression was used for normalization, since we have validated this gene as showing stable expression under our experimental conditions (Cabezas et al., 2018; Pardo-Esté et al., 2018).

### MPOe Activity Quantification

To determine the activation of BMDNs infected with each bacterial strain separately we measured the enzymatic activity of MPO using the Neutrophil Myeloperoxidase Activity Assay Kit (Cayman Chemical). Briefly, infected BMDNs were incubated and at 1 and 3 hpi the color intensity of the 3,3′,5,5′-tetramethyl-benzidine (TMB), which is proportional to the amount of MPO in the sample and is detectable at 650 nm, obtaining the enzymatic activity, in μmoles/min./ml. To obtain the enzymatic specific activity, the results were normalized to the total protein concentration in the samples. Negative control of non-infected neutrophils and free bacteria were used for normalization, in addition to the negative controls with MPO inhibitor (4-aminobenzhydrazide) provided by the kit.

### Protein Carbonylation, Lipid Peroxidation, and Total Glutathione Determination

As indicators of oxidative damage, we determined protein carbonylation, lipid peroxidation, and total glutathione using the Protein Carbonyl Colorimetric, TBARS, and Glutathione Assay Kits (Cayman Chemical), respectively. Briefly, we infected BMDNs with *S.* Typhimurium 14028s and Δ*arcA* separately, as described above. For protein carbonylation, we measured the absorbance at a wavelength of 375 nm of the hydrazone formed by the reaction between 2,4-dinitrophenylhydrazine and the target molecule to determine the concentration (nmol/ml) of protein carbonyls. For TBARS, we used an excitation wavelength of 530 nm and an emission wavelength of 550 nm, and lipid peroxidation was determined as the malondialdehyde concentration (μM) obtained. For glutathione, we used a 410 nm wavelength to read the microplate and obtain the rate of 5-thio-2-nitrobenzoic acid production, which is directly proportional to the concentration of total glutathione (μM). Measurements for each indicator were performed at 1 and 3 hpi. In all cases, negative controls of non-infected neutrophils and free bacteria were used for normalization.

### Statistical Analyses

Gene expression (qRT-PCR) and detection of oxidative damage indicators from the mutant strain were calculated relative to the wild type strain. Gene-by-gene comparisons were performed as individual experiments for each time point using one-way ANOVA with α = 0.05 with the Bonferroni correction, comparing mutant strains with a wild-type strain separately at 1 and 3 hpi using GraphPad 5.01 (Prism1). Additionally, the R package pheatmap was used for results visualization (Kolde, 2015). Correlation analyses were performed using the R package Corrplot 0.84 (Wei and Simko, 2017).

## Results

### ArcA Participates in the *S.* Typhimurium Response to NaOCl Stress by Regulating Bacterial Metabolism

**FIGURE 1:**
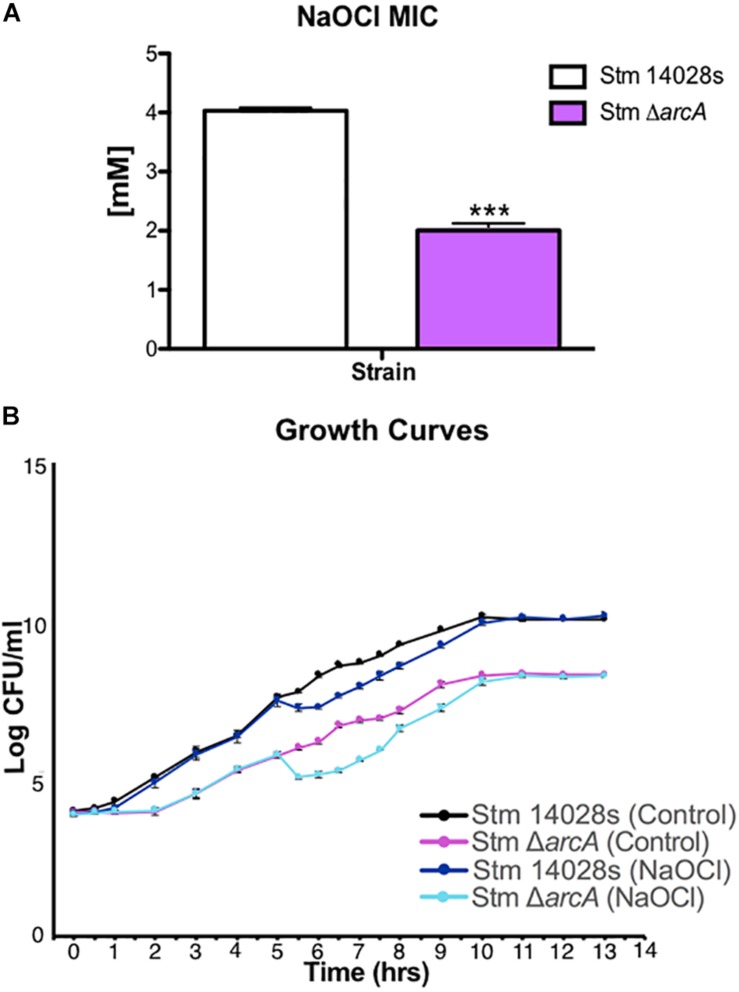
*Salmonella* Typhimurium growth rate in response to NaOCl. **(A)** Minimal inhibitory concentrations (MIC) for the parental 14028s and Δ*arcA* mutant strains. *t-*test (^∗∗∗^*P* < 0.001). Values represent the average of three independent experiments ± SD each with three technical repeats. **(B)** Growth curves for the 14028s and Δ*arcA* mutant strains with and without 1 mM NaOCl treatment.

To understand the role of ArcA during HOCl-related stress *in vitro* under conditions that could mimic those found in neutrophils, we sought to perform a transcriptomic analysis of the parental and Δ*arcA* mutant strains. We used NaOCl for these experiments since the sodium form of HOCl is more stable and dissociates into HOCl in solution (Fukuzaki, 2006). To investigate the optimal concentration of NaOCl in which both Δ*arcA* and the parental strain had similar growth rates, we first determined the minimal inhibitory concentration (MIC) of NaOCl for both strains (Figure 1A). With this information, strains grown to mid-log phase (OD_600_ of 0.4) were exposed to a sublethal NaOCl concentration of 1 mM corresponding to half of the MIC for Δ*arcA*. The growth rates of-Δ*arcA* and the parental strain under this condition were similar, and also the mutant could recover after the addition of NaOCl (Figure 1B).

Next, we obtained transcriptomic datasets from parental and Δ*arcA* treated and untreated with 1 mM NaOCl, as explained in the Materials and Methods, which were mapped against the reference *S.* Typhimurium 14028S (GenBank: GCA_000022165.1). Our transcriptomic data covers 94.4% and 93.8% of the total *Salmonella* ORFs in the Δ*arcA* and parental strain, respectively (Supplementary Table S2). After matching the ORFs that change their expression (after FDR ≤ 0.1 and FC ± 2 filters) between Δ*arcA* (in response to NaOCl relative to the untreated control) and the parental strain (in response to NaOCl relative to the untreated control) to identify the unique ORFs that are dependent on ArcA regulation (ORFs that in the Δ*arcA* strain respond to NaOCl treatment but are not common with those found in the parental strain under stress). The estimated expression levels of these ORFs in the mutant strain suggest that ArcA acts predominantly as a repressor, since 60 ORFs showed higher expression levels, while the remaining 13 had reduced expression.

**FIGURE 2:**
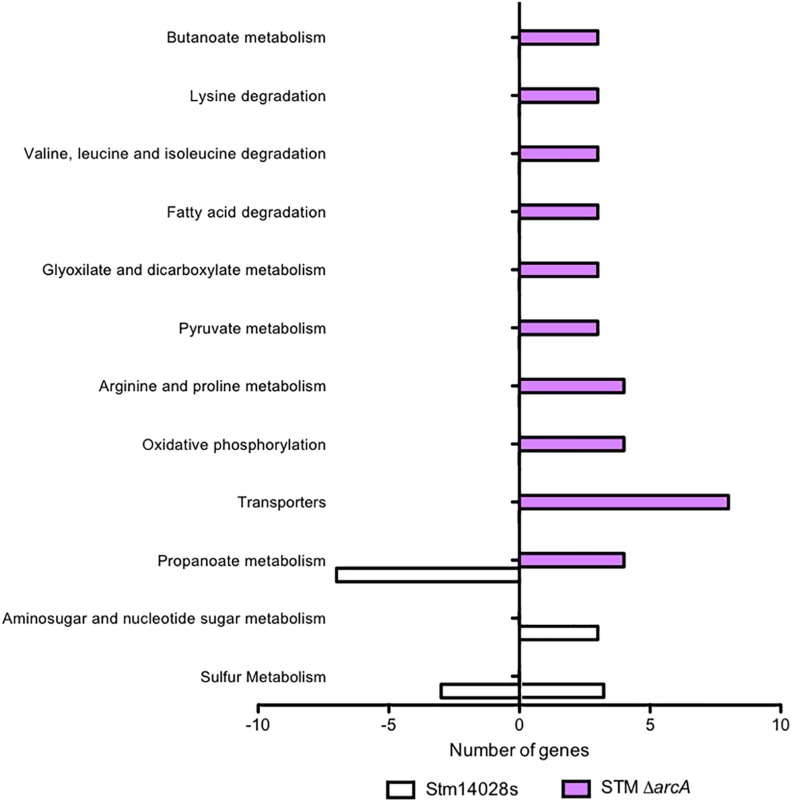
Differential global gene expression in response to 1 mM NaOCl. KEGG pathway enrichment analyses with the differentially expressed genes, comparing the 14028s strain and the Δ*arcA* mutant. White bars show the expression on the 14028s and lavender bars show the expression on the mutant strain normalized by the control condition of the 14028s strain grown in LB medium.

A gene-by-gene comparison revealed different gene expression patterns between the Δ*arcA* mutant and the *S.* Typhimurium parental in response to NaOCl treatment. Only the expression of four genes was shared between the parental strain and Δ*arcA*, while the expression of 82 and 76 genes was unique to Δ*arcA* and the parental strain, respectively. These data suggest that loss of *arcA* caused dysregulation of a different gene set in response to NaOCl. KEGG pathway enrichment analyses revealed that an important part of the transcriptional response to NaOCl mediated by ArcA corresponds to genes involved in metabolism (Figure 2).

**FIGURE 3:**
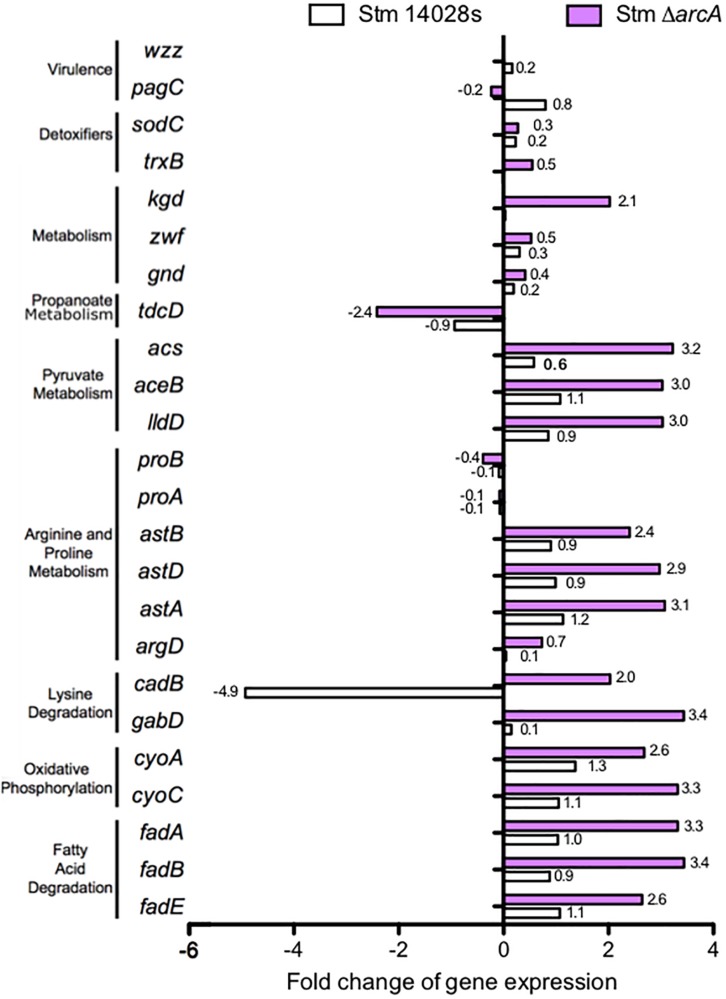
Comparison of differentially expressed genes between the *S.* Typhimurium 14028s parental strain and the Δ*arcA* mutant, both treated with 1 mM of NaOCl relative to the control LB medium.

By calculating the fold change expression of genes belonging to these pathways, as well as for genes encoding proteins with functions related to ROS-resistance, we observed a marked difference in expression levels on several of these genes as a consequence of the mutation on *arcA* (Figure 3). KEGG pathway enrichment analysis revealed that ArcA regulates genes involved in several metabolic pathways such as fatty-acid degradation, oxidative phosphorylation, lysine degradation, arginine-proline metabolism, pyruvate metabolism, and propanoate metabolism. We conclude that ArcA is involved in the bacterial adaptation to ROS by modulating the metabolic fingerprint to effectively reduce oxidative damage and ensure survival under ROS conditions.

### ArcA Mediates Bacterial ROS Resistance Inside Neutrophils, and the Participation of ArcA in Gene Expression Regulation Under HOCl Stress Is Consistent in All Conditions Studied

We used the transcriptome information in response to HOCl *in vitro* to evaluate the transcriptional profile of Δ*arcA* and parental bacteria inside neutrophils. The expression of genes representing the most relevant metabolic pathways found in response to NaOCl *in vitro* was compared by qRT-PCR to that of the same genes in bacteria harvested from infected neutrophils. For these experiments, gene expression was evaluated at 1 and 3 hpi, since these are the times of maximal oxidative burst and also when the *Salmonella-*containing vacuole (SCV) reaches maturation (Goldman, 1990; Eriksson et al., 2000; Vazquez-Torres et al., 2000).

**FIGURE 4:**
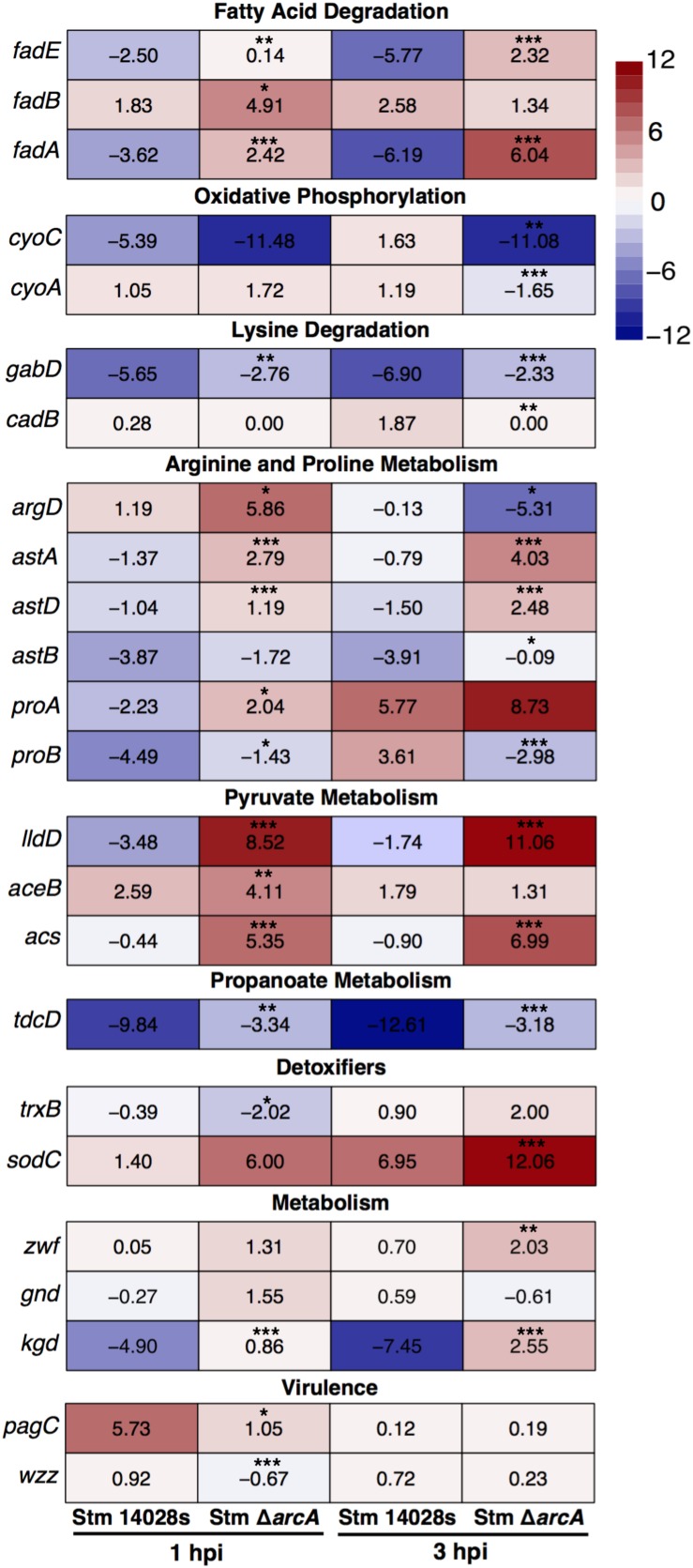
Transcriptional expression of selected genes in Δ*arcA* relative to parental *S*. Typhimurium 14028s in BMDNs at 1 and 3 hpi. Panels show gene expression levels in each strain according to the heatmap scale. Expression data by qRT-PCR were normalized against the expression of *talB* (Cabezas et al., 2018; Pardo-Esté et al., 2018). Values represent the average of three independent experiments; three technical replicates per experiment. One-way ANOVA with the Bonferroni correction (^∗^*P* < 0.05, ^∗∗^*P* < 0.01, ^∗∗∗^*P* < 0.001).

The results show that ArcA negatively regulated fatty acid and lysine degradation, as well as arginine, proline, pyruvate, and propanoate metabolism. This is consistent with a study determining that aspartate, proline, and asparagine are available in the SCV in macrophages (Popp et al., 2015). Also, ArcA positively regulated oxidative phosphorylation (Oxphos) and the antiporter lysine/cadaverine *cadB* gene inside neutrophils (Figure 4). These results suggested the use of extracellular nutrients as TCA cycle intermediaries for the generation of ATP through Oxphos, since other classical pathways are inhibited in intracellular *Salmonella* (Garcia-Gutierrez et al., 2016). Therefore, we conclude that ArcA has a role in maintaining metabolic homeostasis intracellularly by modulating a similar metabolic gene set as seen *in vitro*.

We also investigated the expression of genes encoding proteins required for bacterial intracellular survival in phagocytes (Figure 4). The results demonstrated that during neutrophil infection, ArcA promotes the expression of *trxB* (involved in the repair of disulfide bonds, which are damaged by HOCl) (Gray et al., 2013). Also, expression of the *sodC* gene, which codes for a superoxide dismutase, is negatively regulated inside neutrophils at 3 hpi (Figure 4) but is required in response to HOCl bolus treatment; this could be a manifestation of the gradual production of ROS in the phagosome, contrary to the direct attack of the toxic compound *in vitro* that does not allow the bacteria to adapt efficiently, but rapidly responds to ROS-related stress. Also, ArcA upregulates virulence-related genes not classically associated with antioxidant responses, such as *pagC* and *wzz* (Figure 4). *pagC* encodes a virulence protein involved in intramacrophage survival (Miller, 1991). *wzz* encodes a protein involved in the regulation of O-antigen polysaccharide chain length (Batchelor et al., 1991; Whitfield and Trent, 2014), which is also required for full *Salmonella* virulence (May and Groisman, 2013).

Inside neutrophils, ArcA also repressed the expression of *kgd* (encoding α-ketoglutarate decarboxylase, an enzyme that participates in the TCA cycle) (Figure 4). This is consistent with previous evidence that *Salmonella* does not require an intact TCA cycle to survive intracellularly, as levels of metabolites related to glycolysis, the pentose phosphate pathway, and the TCA cycle decrease in bacteria under stress (Bowden et al., 2009, 2010; Jozefczuk et al., 2010; Garcia-Gutierrez et al., 2016).

**FIGURE 5:**
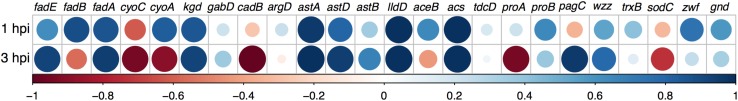
Correlation index between gene expression found *in vitro* with NaOCl treatment (RNAseq) and in intracellular bacteria (qRT-PCR). Data obtained from RNA-seq in response to 1 mM of NaOCl, and gene expression in bacteria harvested from neutrophils evaluated by qRT-PCR at 1 and 3 hpi. Positive correlations are displayed in blue, and negative correlations in red. Color intensity and circle size are proportional to the correlation coefficients relative to RNA-seq values.

We found a positive correlation between the genes expressed under 1 mM of NaOCl treatment *in vitro* and those expressed in bacteria harvested from infected neutrophils at 1 and 3 hpi (Figure 5). Only three genes, *sodC, cadB*, and cyoC, showed negative correlations between their expression levels under 1 mM of NaOCl and in qRT-PCR from bacteria harvested from infected neutrophils. This result was expected, as the conditions inside the neutrophils vary in many aspects compared to the *in vitro* conditions. Bacteria grown in aerobiosis in LB broth with NaOCl bolus treatment negatively regulate oxidative phosphorylation (Oxphos)-related genes, while, on the contrary, their expression is promoted inside neutrophils. Garcia-Gutierrez et al. (2016), demonstrated that under such conditions, *Salmonella* can rely on substrate-level phosphorylation (SLP) and/or Oxphos to fulfill ATP requirements. Also, *sodC* expression is differential, as superoxide concentration varies greatly between the conditionsevaluated, in particular as it is one of the substrates used to generate other ROS inside neutrophil phagosomes.

The other gene with differential expression in both conditions is *cadB*, a transporter related to pH maintenance. Its expression is required inside neutrophils but is not activated *in vitro*, as the conditions in regards to pH change considerably. In particular, neutrophils maintain a near-neutral pH for maintenance of their secreted enzymes, and it has been demonstrated that *Salmonella* is able to sense and acidify in macrophages acidic phagosomes (Chakraborty et al., 2017). Our results suggest that it could also modulate the intraphagosomal pH in neutrophils.

### ArcA Participates in the Defense and Resistance *S.* Typhimurium to Oxidative Damage

**FIGURE 6:**
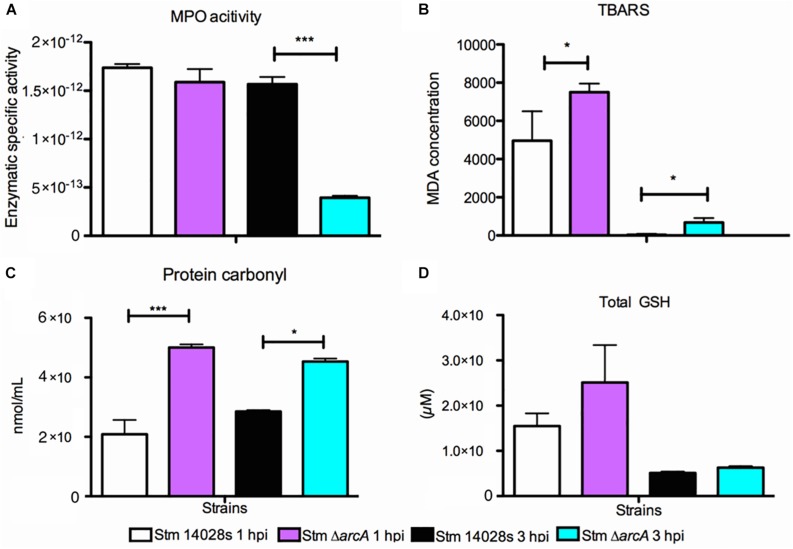
Myeloperoxidase activation and oxidative damage found in *Salmonella* Typhimurium 14028s and Δ*arcA* infecting BMDNs at 1 and 3 hpi. **(A)** Myeloperoxidase enzyme-specific activity in BMDNs infected with *S*. Typhimurium 14028s and Δ*arcA*. Measured as the protein units (μmol/ml) normalized by total protein concentration. **(B)** Thiobarbituric acid reactive substances (TBARS) as an indicator of oxidative damage in Δ*arcA* relative to *S*. Typhimurium 14028s harvested from infected BMDNs at 1 and 3 hpi. **(C)** Protein carbonylation as an indicator of oxidative damage in Δ*arcA* relative to *S*. Typhimurium 14028s harvested from infected BMNs at 1 and 3 hpi. **(D)** Glutathione as an indicator of cellular redox state in Δ*arcA* relative to *S*. Typhimurium 14028s harvested from infected BMDNs at 1 and 3 hpi. One-way ANOVA with the Bonferroni correction (^∗^*P* < 0.05, ^∗∗^*P* < 0.01, ^∗∗∗^*P* < 0.001). Values represent the average of five independent experiments ± SD, three technical replicates per experiment.

Since ArcA regulates several important genes in response to ROS inside neutrophils, we determined the MPO activity in infected neutrophils at the same times post-infection used for transcriptional analyses, which provides an estimation of the HOCl production. The results indicate that MPO activity is detectable at similarly high levels in neutrophils infected with either Δ*arcA* or the parental strain at 1 hpi (Figure 6A). In contrast, at 3 hpi, MPO activity decreases by up to 75% in neutrophils infected with Δ*arcA* compared to those infected with the parental strain (Figure 6A). The lower MPO activation suggests that neutrophils infected with bacteria lacking *arcA* are more susceptible to oxidative damage and therefore killed more rapidly.

Lipid peroxidation, protein carbonylation, and total glutathione are indicators of oxidative damage in bacteria. The Δ*arcA* mutant shows a greater amount of oxidative damage in lipids and proteins as measured by lipid peroxidation and protein carbonylation (Figures 6B,C). However, the cellular redox state, determined as the amount of total glutathione, remains similar in all strains (Figure 6D). There is a positive correlation between MPO activity and increased lipid peroxidation (Morales et al., 2012; Drazic et al., 2015; Okada et al., 2016) and protein carbonylation (Levine et al., 1990; Shacter et al., 1994; Wilkie-Grantham et al., 2015; Beavers and Skaar, 2016). These results further support the notion that ArcA plays a pivotal role in oxidative damage resistance. Indeed, mutant bacteria activate significantly less MPO in neutrophils and display a higher amount of oxidative-associated damage.

## Discussion

Neutrophils play an important role in the innate immune response to *S*. Typhimurium infection (Conlan, 1997) in particular by producing HOCl, a short-lived toxic compound that rapidly targets and damages key macromolecules (Davies, 2005). The transcription factor ArcA is required for bacterial survival in epithelial cells, macrophages, and neutrophils by regulating detoxification-, membrane permeability-, and virulence-related genes inside macrophages and neutrophils (Pardo-Esté et al., 2018). In this work, we show that ArcA is also relevant in the metabolic fingerprint of *S.* Typhimurium in response to HOCl. This was revealed by a clear dysregulation of the transcriptomic profile under NaOCl treatment in the absence of *arcA* and by showing that ArcA also influences the metabolic adaptation of bacteria upon phagocytosis by neutrophils.

In this study, we evaluated three very specific conditions concerning HOCl concentrations, namely bolus treatment and 1 hpi and 3 hpi in neutrophils, and determined that the participation of ArcA in these conditions is similar. Further, there was a predominantly positive correlation between expression patterns from bacteria treated with sub-lethal concentrations of NaOCl *in vitro* and those found in bacteria at 1 and 3 hpi in infected neutrophils. We have previously reported that, at these hpi times, infected neutrophils produce increasing amounts of HOCl, reaching up to 200 μM at 3 hpi (Pardo-Esté et al., 2018).

*Salmonella* Typhimurium metabolism is very flexible, and T3SS1 expression is also influenced by metabolism. For example, cell invasion increases when pyruvate metabolism is abolished (Abernathy et al., 2013). Here, we determined that ArcA inhibits genes related to pyruvate metabolism (Figure 4). Additionally, intracellular *S*. Typhimurium might fulfil its ATP requirements via substrate-level phosphorylation (SLP) and/or Oxphos (Garcia-Gutierrez et al., 2016), and TCA cycle enzymes do not appear to be essential inside macrophages (Bowden et al., 2009, 2010; Garcia-Gutierrez et al., 2016). Our results agree with this notion, several metabolic pathways are inhibited by ArcA in bacteria inside neutrophils, while genes related to Oxphos are upregulated. This indicates that the ability of the pathogen to resist HOCl-related stress is associated with its capacity to metabolically adapt and use the available metabolites to withstand oxidative damage.

We have determined that ArcA negatively regulates pyruvate metabolism, fatty acid degradation, and at least part of the TCA cycle in response to HOCl-related stress and oxygen availability inside neutrophils (Figure 4). Evidence shows that *Salmonella* does not use the β-oxidation pathway during RAW 264.7 macrophage infection in DMEM medium with high levels of glucose (Diacovich et al., 2017). During infection, the bacterium allocates resources very tightly, especially to protein production and pathway usage. As sugars are the primary carbon sources of *S.* Typhimurium, here we demonstrate that ArcA represses the β-oxidation pathway in favor of sugar uptake and further catabolism in response to oxidative stress. The intracellular redox state also requires a balanced pool of (NADH/NAD^+^), as well as the conversion of pyruvate to acetyl-CoA, which finally fuels the TCA cycle. Thus, the pyruvate node is essential in regulating both the cofactor ratio and the rate of carbon conversion within the TCA cycle. These results confirm that ArcA controls the redox state of the cell, as it is involved in regulating at least part of the TCA cycle. ArcA also appears to govern the metabolism of various amino acids, such as proline, arginine, lysine, and threonine, inside neutrophils (Figure 4). The proline and arginine biosynthesis pathways require ATP and NADP(H) for their synthesis. The latter cofactor is critical to counteract oxidative damage and is also required for glutathione synthesis.

*Salmonella* Typhimurium can utilize amino acids obtained from the host during intracellular life (Das et al., 2010), enabling an efficient mechanism for intracellular proliferation. In fact, intracellular *S.* Typhimurium utilizes medium-derived amino acid rather than host cell-derived amino acids in an SPI-2-dependent manner (Popp et al., 2015), a bacterial strategy for host cell endosomal transport for intracellular nutrition. In agreement with this notion, we found the antiporter of lysine/cadaverine *cadB* to be upregulated by ArcA (Figure 4), which would allow a continuous amino acid supply from the host cell’s cytoplasm of the host cell into the bacteria-containing vacuoles which is key for survival and, at the same time, limiting, because hypochlorite exposure causes methionine and alanine to significantly decrease in treated cells (Drazic et al., 2015).

Further, the use of oxidative phosphorylation for energy production inside the vacuole is suggested by the upregulation of *cyoC* and *cyoA* (Figure 4), indicating that the environment *in vivo* is somewhat anaerobic and requires oxidative phosphorylation for ATP generation. To obtain the NAD and FADH required for Oxphos, the bacteria under these conditions might be using amino acids obtained from the host cell (Popp et al., 2015). In addition to energy sources, intracellular *S.* Typhimurium requires other virulence traits involved in manipulating the host cell (Holden, 2002; Fabrega and Vila, 2013; Hurley et al., 2014).

One unexpected finding was that the MPO activity was lower in Δ*arcA*-infected neutrophils compared to neutrophils infected with the parental strain. Despite this, neutrophils cause more oxidative damage to the bacterial mutant than to the parental strain. We speculate that the loss of ArcA may affect the mechanism of MPO activation directly or indirectly, thus explaining the lower enzymatic activity, but this observation will require additional experimentation.

Collectively, our results indicate that the transcription factor ArcA is required for the resistance of *S.* Typhimurium to HOCl-related stress in general and, in particular, to the conditions found inside neutrophils. This response regulator modulates the gene expression of metabolic pathways that enable the bacteria to obtain ATP and also controls some additional virulence- and repair-related genes, which together provide resistance to oxidative damage during the pathogen’s infection cycle. Therefore, ArcA-mediated modulation of metabolic homeostasis contributes to the virulence repertoire that *Salmonella* uses to evade antimicrobial strategies in the host.

## Data Availability Statement

The datasets generated for this study can be found in the NCBI SRA database under accession numbers SRR9188680 and SRR9188681 (Bioproject PRJNA357075).

## Ethics Statement

The animal study was reviewed and approved by the Bioethics Committee of Universidad Andrés Bello, Protocol 06/2016 in the framework of FONDECYT Grant #1160315.

## Author Contributions

CS and CP-E conceived and designed the study. CP-E, JC-S, GK, CA, and YS carried out experiments. CC, AB, and MB extracted *in vitro* RNA samples. NM, AH, CM, IP-C, EC-N, and MV contributed with the reagents, materials, and analysis tools. CP-E, JC-S, IP-C, MV, and CS wrote the manuscript. All authors read and approved the final manuscript.

## Conflict of Interest

The authors declare that the research was conducted in the absence of any commercial or financial relationships that could be construed as a potential conflict of interest.
